# Development and validation of an RNA-seq-based transcriptomic risk score for asthma

**DOI:** 10.1038/s41598-022-12199-0

**Published:** 2022-05-23

**Authors:** Xuan Cao, Lili Ding, Tesfaye B. Mersha

**Affiliations:** 1 Division of Statistics and Data Science, Department of Mathematical Sciences, University of Cincinnati, Cincinnati, OH USA; 2 Division of Biostatistics and Epidemiology, Department of Pediatrics, Cincinnati Children’s Hospital Medical Center, University of Cincinnati, Cincinnati, OH USA; 3 Division of Asthma Research, Department of Pediatrics, Cincinnati Children’s Hospital Medical Center, University of Cincinnati, Cincinnati, OH USA

**Keywords:** Gene expression, Asthma, Predictive markers

## Abstract

Recent progress in RNA sequencing (RNA-seq) allows us to explore whole-genome gene expression profiles and to develop predictive model for disease risk. The objective of this study was to develop and validate an RNA-seq-based transcriptomic risk score (RSRS) for disease risk prediction that can simultaneously accommodate demographic information. We analyzed RNA-seq gene expression data from 441 asthmatic and 254 non-asthmatic samples. Logistic least absolute shrinkage and selection operator (Lasso) regression analysis in the training set identified 73 differentially expressed genes (DEG) to form a weighted RSRS that discriminated asthmatics from healthy subjects with area under the curve (AUC) of 0.80 in the testing set after adjustment for age and gender. The 73-gene RSRS was validated in three independent RNA-seq datasets and achieved AUCs of 0.70, 0.77 and 0.60, respectively. To explore their biological and molecular functions in asthma phenotype, we examined the 73 genes by enrichment pathway analysis and found that these genes were significantly (p < 0.0001) enriched for DNA replication, recombination, and repair, cell-to-cell signaling and interaction, and eumelanin biosynthesis and developmental disorder. Further in-silico analyses of the 73 genes using Connectivity map shows that drugs (mepacrine, dactolisib) and genetic perturbagens (PAK1, GSR, RBM15 and TNFRSF12A) were identified and could potentially be repurposed for treating asthma. These findings show the promise for RNA-seq risk scores to stratify and predict disease risk.

## Introduction

Genome-wide association studies (GWAS) have been used to identify individual variants influencing disease risk^1^. However, most complex diseases are influenced by several loci, each with a small effect on its own, and polygenic approaches that group individual variants collectively influence a phenotypic trait offer a more predictive value than is possible by single variant approaches^2^. The most popular polygenic approach is polygenic risk score (PRS). PRS is defined as weighted sums of risk alleles of a pre-specified set of single nucleotide polymorphisms (SNPs)^2,3^. Weights in PRS are typically defined by estimated effect sizes of the SNPs and determined externally from independent studies, but there are also approaches that accommodate cases where appropriate external weights are not available, and internal weights from within the study are adopted instead^4–6^. PRS has proven to be statistically powerful for testing marginal genetic effects^7^ and gene-environment interaction effects^8^, and for predicting risks of complex disease^9^.

Studies are now adapting PRS approaches to transcriptomics data^10–14^. A 20-gene microarray gene expression-based risk score was built to predict the overall survival and risk classification for patients with chronic lymphocytic leukemia^10^. Zhu et al.^13^ developed a microarray expression-based risk score with 16 survival‑associated autophagy‑related genes for prognostic assessment of multiple myeloma. With technological advancement, RNA-seq becomes a more unbiased profiling method for the entire transcriptome than microarray platform. Compared with microarray analysis, RNA-seq can detect novel transcripts, quantify expression over a wider dynamic range, and detect rare and low-abundance transcripts^15–18^.

Asthma has been recognized as a systemic disease consisting of networks of genes showing inflammatory changes that involve a broad spectrum of adaptive and innate immune systems. Utilizing measurable characteristics including gene expression can help to stratify asthma patients and develop strategies to predict asthma severity and risk^19^. Castro-Rodriguez et al.^20^ developed clinical data based asthma predictive index (API). Belsky et al.^21^ derived a PRS based on multi-locus profiling from published GWAS. Recently, our group developed the pediatric asthma risk score (PARS) algorithm that integrates clinical and demographic factors^22^. PARS showed improvement compared with previous tools such as API^23^. However, both are based on clinical data and did not incorporate biological information including transcriptomic data.

The objectives of this study were to uncover differentially expressed signature genes between asthmatic and healthy controls using RNA-seq data and to identify an optimal subset of signature genes to construct a RNA-seq-based risk score (RSRS) that allows the accommodation of demographic factors including age and gender. The performance of RSRS was evaluated in an independent RNA-seq dataset. Additionally, we explored the DEGs in various publicly available resources such as asthma GWAS catalog and further determine their biological roles with enrichment analysis. Finally, we applied Connectivity Map (CMap) analysis approach to explore potential drug targets by systematically mining functional connections between asthma, RSRS, and perturbagens^24^.

## Materials and methods

**Figure 1:**
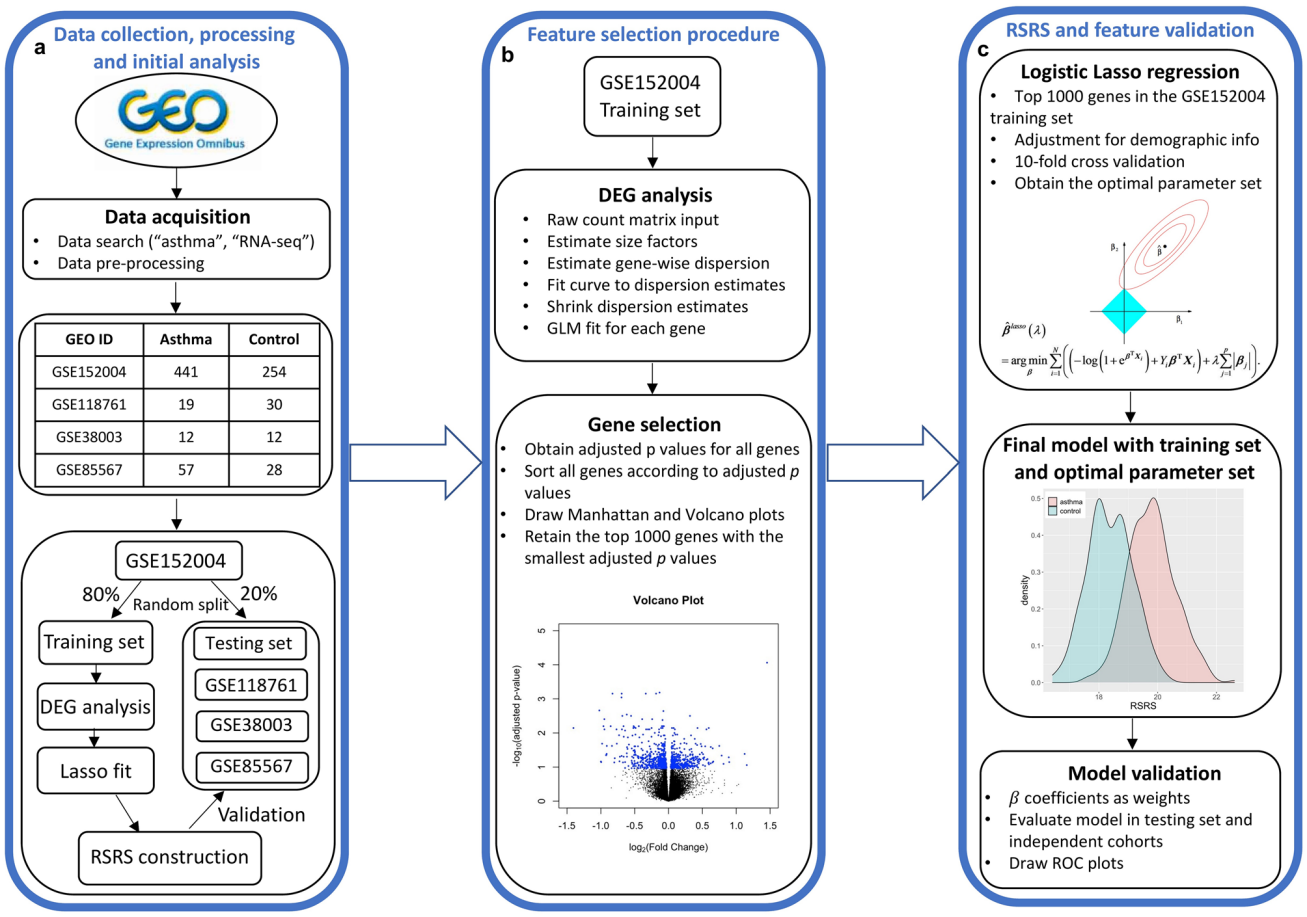
Study workflow for constructing the RSRS containing the steps of data acquisition and analysis. (**a**) Public data collection, processing and initial data analysis; (**b**) feature selection pipeline including DEG analysis and gene selection; (**c**) RSRS formulation and model validation in the testing set and independent cohorts.

To construct an RSRS for asthma, RNA-seq datasets were obtained from two independent studies^25,26^ downloaded from the publicly available NCBI GEO (Gene Expression Omnibus, NCBI) database. Individuals in discovery dataset were randomly split into training and testing sets. DEGs were determined in the training set according to genome-wide adjusted p values. The selected DEGs were used to construct the RSRS in training dataset using logistic Lasso regression and develop a prediction model. The final model was tested in the testing set and validated in the other independent datasets. Datasets and analysis steps were summarized in Fig. 1.

### RNA sequencing (RNA-seq) datasets

Eligible GEO RNA-seq asthma datasets were selected based on the following inclusion criteria: (a) the dataset must compare asthma patients to non-asthma controls, and (b) the dataset must be generated from same tissue type. Four asthma RNA-seq studies (accession: GSE152004, GSE118761, GSE38003 and GSE85567)^25–28^ fulfilled the inclusion criteria were used in the subsequent analysis. The following information was extracted from each study: (1) GEO accession numbers, (2) asthma and healthy status of the samples, (3) demographic information, and (4) count data from sequencing reads. In addition, demographic information including age and gender was obtained for GSE152004 and GSE118761. The raw count datasets were downloaded from GREIN (GEO RNA-seq Experiments Interactive Navigator)^29^. There were 695 individuals in the GSE152004 dataset including 441 asthmatic subjects and 254 non-asthmatic subjects^26^. RNA-seq data in GSE152004 was used for discovery of DEGs and model training and testing, while GSE118761 with 19 asthma subjects and 30 controls^25^, GSE38003 with 12 asthmatics and 12 controls^28^, and GSE85567 with 57 asthmatics and 28 controls^27^ were used for model validation. The statistical software R was used to extract the expression values of individual genes and demographic information of different samples in asthma and control groups.

#### Data pre-processing

Normalized datasets were download from GREIN (GEO RNA-seq Experiments Interactive Navigator)^29^, which uses the normalization method of Trimmed Mean of M-values (TMM) as implemented in the Bioconductor package edgeR^30^. DESeq2 R package was used to parse RNA-seq data and store the read counts in the form of a matrix of integer values^31^. Pre-filtering was performed to keep only genes that have at least 10 reads total.

### RNA-seq-based risk score (RSRS) development and validation

The RNA-seq data GSE152004 was randomly split into 80% as training and 20% as testing while maintaining the same asthma: control ratio. DEG selection and risk score development were solely based on the training set. Three independent RNA-seq asthma datasets (GSE118761, GSE38003 and GSE85567) were used for model validation.

#### Constructing RNA-seq-based risk score (RSRS)

Analogous to the polygenetic risk score (PRS)^32,33^, RSRS of the $i$th individual was constructed as weighted sum of the individual’s transformed and normalized RNA-seq expression values of $K$ identified genes (${g}_{i1}^{\mathrm{log}}, {g}_{i2}^{\mathrm{log}}, \dots , {g}_{iK}^{\mathrm{log}})$,

$$RSR{S}_{i}={\beta }_{1}{g}_{i1}^{log}+{\beta }_{2}{g}_{i2}^{log}+\dots +{\beta }_{K}{g}_{iK}^{log}.$$

Here, ${\beta }_{k}, k=1, 2, \dots , K$ denotes weights and ${g}_{ik}^{\mathrm{log}}=\mathrm{log}({g}_{ik}+1)$ for $i=\mathrm{1,2},\dots ,n$ and $k=\mathrm{1,2},\dots ,K$ denotes normalized and log-transformed RNA-seq expression values. As the normalized counts could contain null values, we shifted them by one before log-transforming.

The log-transformed RNA-seq gene expression values are typically distributed more symmetrically than before transformation and have fewer extreme values compared to the untransformed data^34^.

#### Variable selection for RSRS

To identify the signature genes to be included in the RSRS and estimate the corresponding weights of those genes, DEG analysis will be conducted on the training data to select a candidate set of DEGs, followed by Lasso logistic regression for further variable selection and weight estimation.

##### DEG analysis

Differential expression analysis of the training set was conducted using the DESeq2 R package. We extracted DEG results including log2 fold changes, p values and adjusted p values for all genes, where the p values were attained by the Wald test and corrected for multiple testing using the Benjamini and Hochberg method^35^. Manhattan plot of the genome-wide DEG analysis results was composed via the ggbio R package^36^, where genes were annotated using the biomaRt R package from the Bioconductor project and mapped to corresponding chromosome locations^37,38^.

##### Lasso logistic regression

The 1000 genes with the smallest adjusted p values in training set, as well as demographic factors such as age and sex, were included in Lasso logistic regression to select the optimal subset of DEGs to construct RSRS. To compare with the 1000 gene list, we also considered the DEG list with the top genes ranked by fold change and adjusted p value less than 0.05^39,40^. The same Lasso logistic regression was applied to the resulting gene list. Lasso is a penalized regression approach that performs variable selection and regularization by maximizing the log-likelihood function with the constraint that the sum of the absolute values of the coefficients is less than or equal to some positive constant^41^. Lasso logistic regression was carried out using the glmnet R package^42^ with tenfold cross validation on the training set to select the optimal parameters. Age and sex were included in Lasso logistic regression to control the potential impact of demographic information on the disease risk. Genes with non-zero estimated beta weights were the optimal subset of features used to construct the RSRS.

#### RSRS and prediction model for disease risk

Given the optimal tuning parameters and the optimal subset of DEGs and demographic factors with non-zero beta weights identified in Lasso logistic regression with tenfold cross validation, an RSRS and prediction model for disease risk was developed in the whole training set using a logistic regression model. For the $i$ th individual with a binary disease outcome ${y}_{i}=1, 0$, $i=\mathrm{1,2},\dots ,n,$ we consider the following logistic regression model,

$$\mathrm{log}\left(\frac{\mu }{1-\mu }\right)={\beta }_{0}+RSR{S}_{i}+{{\varvec{X}}}_{{\varvec{i}}}{{\varvec{\beta}}}_{{\varvec{K}}+1}={\beta }_{0}+{{\beta }_{1}g}_{i1}^{log}+{{\beta }_{2}g}_{i2}^{log}+\dots +{{\beta }_{K}g}_{iK}^{log}+{{\varvec{X}}}_{{\varvec{i}}}{{\varvec{\beta}}}_{{\varvec{K}}+1}.$$

Here $\mu$ represents the probability of having the disease, i.e., $\mu =P\left({y}_{i}=1\right)=\frac{\mathrm{exp}\left({\beta }_{0}+RSR{S}_{i}+{{{\varvec{X}}}_{{\varvec{i}}}{\varvec{\beta}}}_{{\varvec{K}}+1}\right)}{1+\mathrm{exp}\left({\beta }_{0}+RSR{S}_{i}+{{{\varvec{X}}}_{{\varvec{i}}}{\varvec{\beta}}}_{{\varvec{K}}+1}\right)}$ and ${\beta }_{0}$ is the intercept. Any demographic factors that had non-zero estimated coefficient in Lasso logistic regression will be included in the model by the term ${{\varvec{X}}}_{{\varvec{i}}}{{\varvec{\beta}}}_{{\varvec{K}}+1}$**,** where ${{\varvec{X}}}_{{\varvec{i}}}$ is the matrix of demographic data, and ${{\varvec{\beta}}}_{{\varvec{K}}+1}$ is the vector of regression coefficients to be estimated from the whole training set. The exponential function ${e}^{{\beta }_{k}}$ of the $k$th regression coefficient ${\beta }_{k}$ is the odds ratio associated with a one-unit increase in the log of the normalized gene expression count for the $k$th gene^43,44^. The estimated regression coefficient $\widehat{{\beta }_{k}} (k=\mathrm{1,2},\dots ,K)$ is the estimated weight for the $k$th gene in the RSRS.

##### Pairwise correlation

To investigate if the *K* signature DEGs retained in the RSRS provide independent information to asthma risk, we visualized the pair-wise Pearson correlation among the normalized and log-transformed gene expression levels of the selected genes by plotting the heat map using the corrplot R package.

##### Model validation

Finally, the prediction model with RSRS and demographic information was both tested in the testing dataset and validated in the independent sample GSE118761. RSRS without age and gender was implemented to predict the disease risk in the independent cohorts GSE38003 and GSE85567. Different prediction models based on the DEG list ranked by fold change with adjusted p value < 0.05, and the top 10, 50, 100 genes ranked by p value were also formulated and compared with the prediction models with RSRS utilizing the genes and demographic factors selected by Lasso. The confidence interval (CI) for the area under the receiver operating characteristics curve (ROC) was deduced based on the covariance matrix derived from generalized U-statistics^45^ using an accelerated algorithm^46^. Both the ROC curves and AUC values were implemented in the R package pROC^47^. AUC was used to compare the models. We used the R package cutpointr^48^ to estimate the optimal cut points that maximizes the Youden-Index^49^ for determining the binary disease outcome and validate performance using bootstrapping.

### Pathway and network analyses of RSRS

Ingenuity pathway analysis (IPA) software (Qiagen, USA) was used to generate putative networks and pathways based on the manually curated knowledge database of pathway interactions. The networks were generated using the genes retained in the RSRS after Lasso logistic regression in both direct and indirect relationships/connectivity. These networks were ranked by scores that measured the probability that the genes were included in the network beyond chance^50^. Canonical pathways associated with input genes were elucidated with a ratio to examine pathway enrichment and statistical significance adjusted for multiple testing. The p value is calculated using a right-tailed Fisher Exact test and indicates the likelihood of the pathway association under the random model.

### Linking asthma RSRS with asthma GWAS catalog datasets

There were 168 asthma studies resulting 2811 SNPs from GWAS Catalog (Hindorff et al. 2009) (accessed January 2022). Inclusion of asthma GWAS catalog-based associations was limited to those studies with p values of less than 5 × 10E−8 (http://www.ebi.ac.uk/gwas/). Overlap between genes from RSRS after Lasso logistic regression and asthma genes from GWAS catalog were examined.

### Connectivity Map (CMap) analysis

Next, we used Connectivity Map (CMap) analysis approach to explore potential drugs targeting asthma by systematically mining functional connections between asthma disease, RSRS, and perturbagens^24^. CMap currently covers > 1309 compounds connected with 7000 expression profiles^51^. This approach can identify drugs that affect the RNA-seq-based risk score and perturbagens that might potentially reverse asthma risk^52^. DEGs retained in the RSRS were divided into two groups, one for upregulated and the other for downregulated group. The CMap analysis was performed through the web interface CLUE (https://clue.io/), a cloud-based platform used to analyze perturbation-driven gene signatures, following a standard protocol described by Wang et al.^53,54^. CMap instance was measured by an enrichment score, which ranged from − 1 to 1, and a permutation p value. Connectivity score of below -0.85 or above 0.85 was considered for this analysis^54^.

## Results

### Gene selection based on DEG analysis

**Figure 2:**
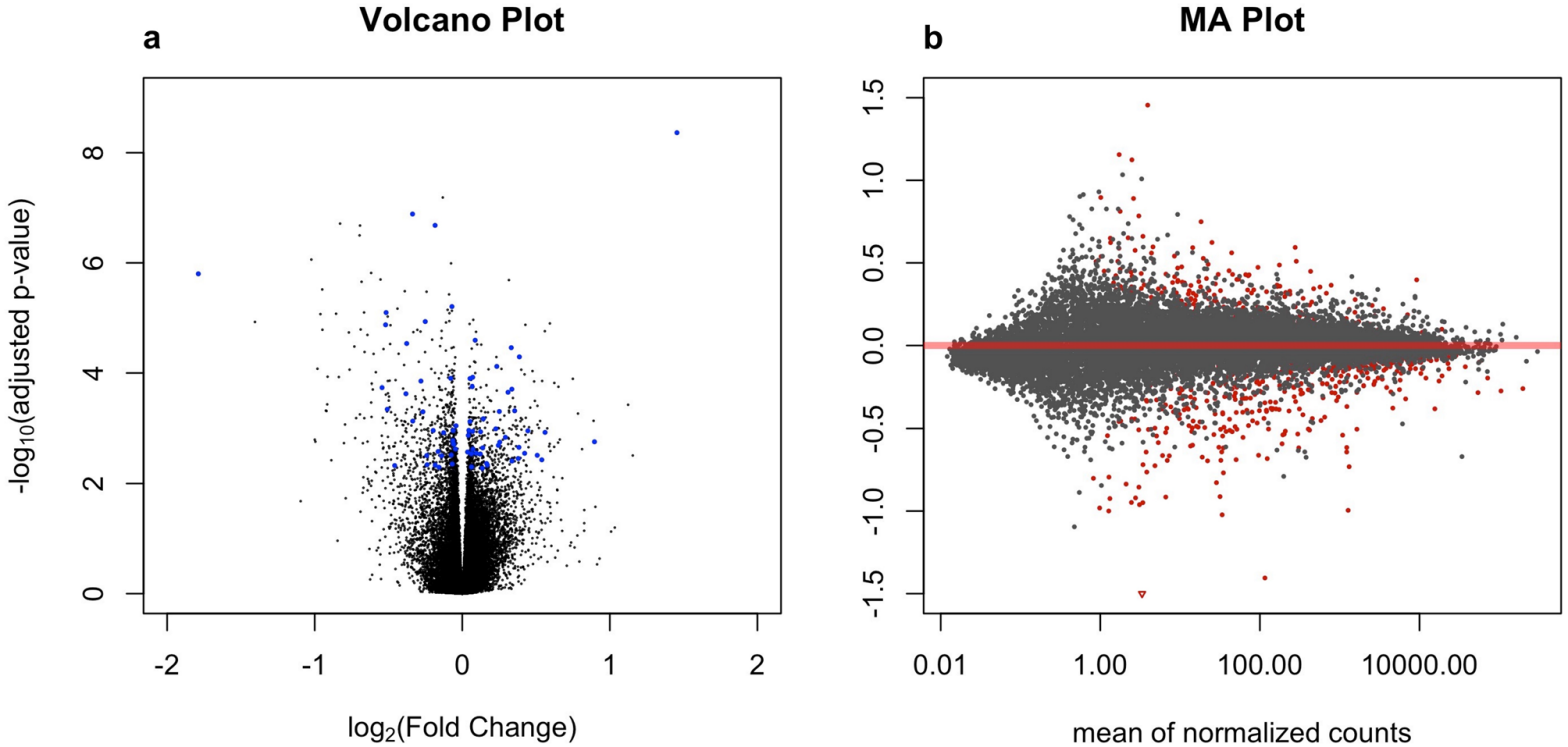
Initial quality checking of the RNA-seq data based on the training set. (**a**) Volcano plot of − log_10_ adjusted p values (on the y-axis) versus log_2_ fold changes (on the x-axis) using the training set. The blue points correspond to the 73 RSRS genes. (**b**) MA plot, which is a scatter plot of log2 fold changes (on the y-axis) versus the mean of normalized counts (on the x-axis), where points were colored red if the genes were selected. Points which fall out of the window were plotted as open triangles pointing either up or down.

Genome-wide DEG analysis results of the training set were visualized using volcano plot and MA plot (Fig. 2). The Manhattan plot of DEGs was given in Fig. S1. We retained 1000 genes with smallest adjusted p values in the training set.

### Predictive value of RSRS

**Figure 3:**
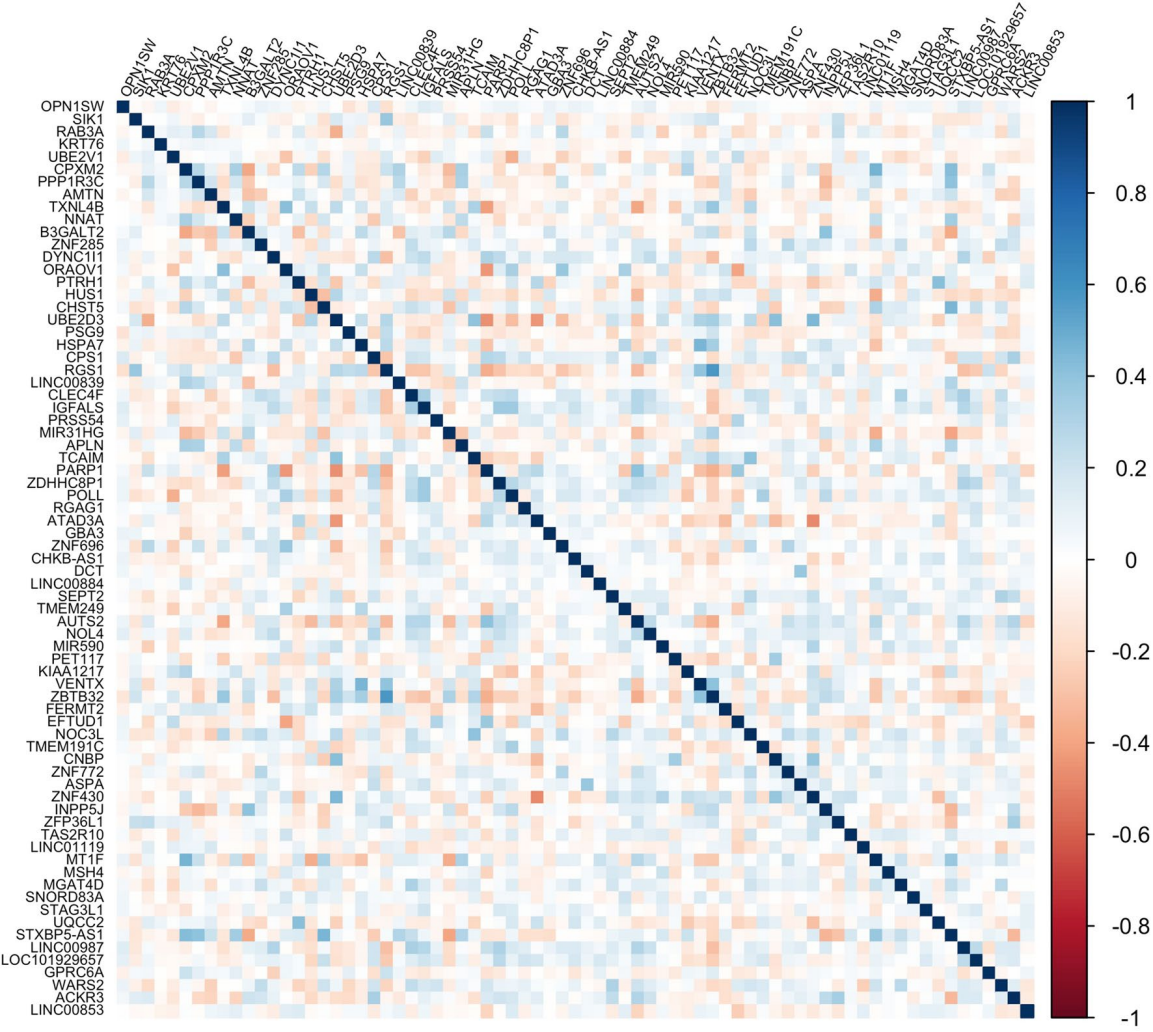
The heat map of the pair-wise Pearson correlation among the normalized and log-transformed gene expression values of 73 genes.

Of the 1000 genes, 73 genes were selected by logistic Lasso regression with tenfold cross validation (Fig. 2a). Weights, log2 fold changes and adjusted p values from logistic regression models of the 73 genes on the whole training set were provided in Table S1. As shown in Table S1, the odds ratios of 33 genes including SIK1, RAB3A, KRT76, UBE2V1 among others are larger than 1. Therefore, these genes can increase the odds and show an up-regulated effect on the outcome of asthma, while 40 genes including CNBP, POLL, ZNF696, HUS1 among others impose a down-regulated effect on the disease response. In Fig. 3, we generated the heat map of the pair-wise Pearson correlation among these 73 genes in the training data and the result of principal component analysis (PCA) was shown in Fig. S2. The results showed that the 73 genes are not correlated and provide independent information to asthma risk. The gender factor also contributed to the disease risk. In particular, girls would be at a higher risk for asthma compared with boys.

**Figure 4:**
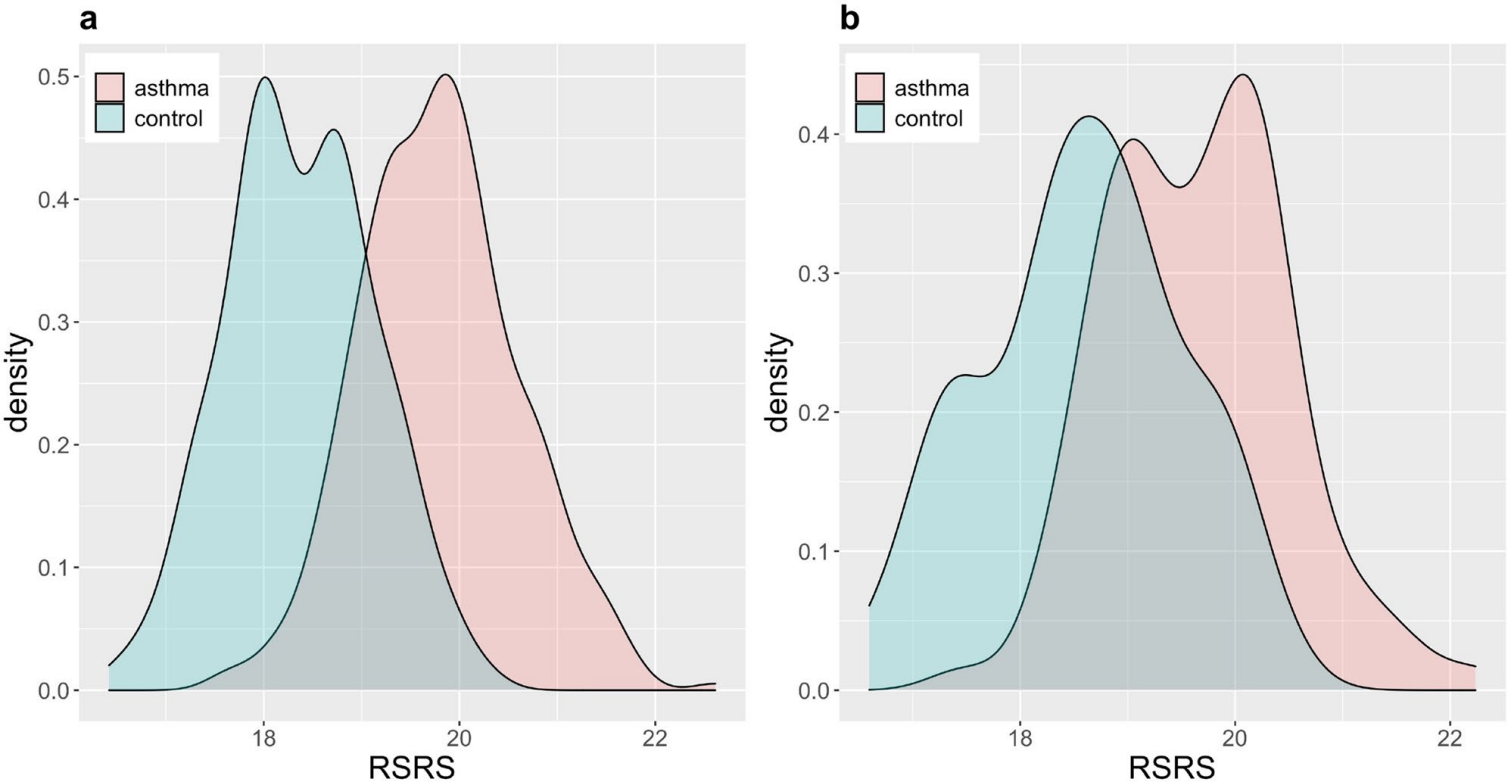
Density plots showing the asthma risk based on RSRS for different groups. (**a**) In training set; (**b**) testing set.

The density distributions of RSRS for asthma vs. healthy controls in both the training and testing sets are depicted in Fig. 4. Note that a higher value of RSRS indicates a higher probability for asthma. For both the asthmatic and control groups, the density distribution resembles mostly a unimodal distribution with thin tails, which indicates only a few individuals have higher probabilities of developing asthma compared with others.

#### Testing set

**Figure 5:**
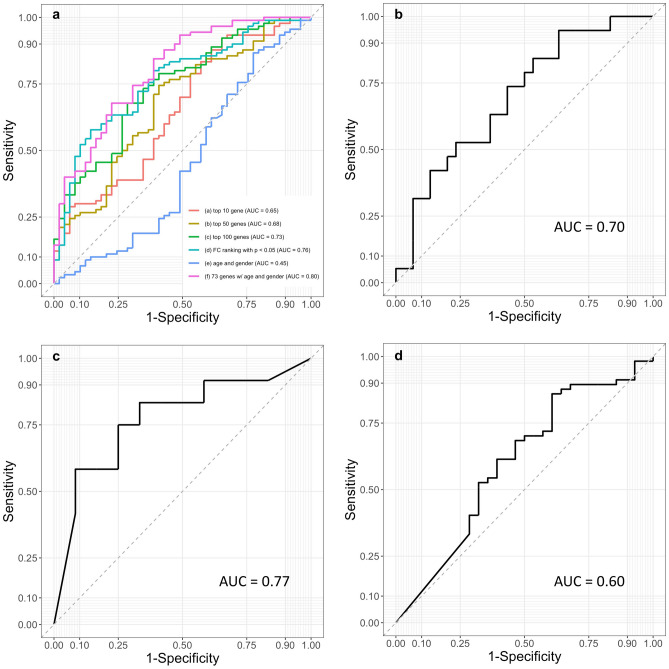
ROC curves for the asthma prediction performance of RSRS. (**a**) testing set and comparison with risk scores based on the DEG list ranked by fold change (FC) with p < 0.05, and the top 10, 50,100 genes ranked by p value; (**b**) in the independent cohort GSE118761 (AUC = 0.70); (**c**) in the independent cohort GSE38003 (AUC = 0.77); (**d**) in the independent cohort GSE85567 (AUC = 0.60).

The ROC curves and AUC values in the testing set corresponding to different risk scores utilizing different numbers of top ranked genes were given in Fig. 5a. The RSRS based on 73 genes with adjustment for demographic information achieved the highest AUC of 0.80 (CI 0.73–0.88) compared with other risk scores based on the DEG list ranked by fold change with adjusted p value < 0.05, and the top 10, 50, 100 genes ranked by p value. The optimal cut point for thresholding the probabilities obtained from the prediction model is 0.54, which yields an accuracy of 0.76. Figure S3 lists various distribution plots for cutoff points and out-of-bag performance based on bootstrapping.

#### Validation dataset

For the independent validation dataset GSE38003, the 73-gene-based RSRS and sex achieved an AUC of 0.70 (95% CI 0.55–0.85) (Fig. 5b). The optimal cut point for thresholding the probabilities yielded an accuracy of 0.61. Figure S4 lists various distribution plots for cutoff points and out-of-bag performance based on bootstrapping for GSE118761. For GSE38003 and GSE85567, the 73-gene-based RSRS achieved AUCs of 0.77 (95% CI 0.58–0.97) (Fig. 5c) and 0.60 (95% CI 0.47–0.74) (Fig. 5d), respectively. The optimal cut point for thresholding the probabilities yielded an accuracy of 0.67 for GSE38003 and an accuracy of 0.60 for GSE85567. Figures S5 and S6 list various distribution plots for cutoff points and out-of-bag performance based on bootstrapping for GSE38003 and GSE85567, respectively.

### Enrichment analysis of RSRS for network and pathways

The biological and molecular functions of the 73 RSRS genes were examined for enriched pathways using the Ingenuity Pathway Analysis system.

Using the Ingenuity Pathway Analysis of the 73 RSRS genes, we found six and five enriched networks and pathways, respectively (score ≥ 2). The functions of the top networks and pathways are shown in Table S2 and Fig. S7. Enriched diseases and functions involve organismal injury and abnormalities, developmental disorder, and connective tissue disorders.

### Overlap of genes between RSRS and asthma GWAS catalog

**Figure 6:**
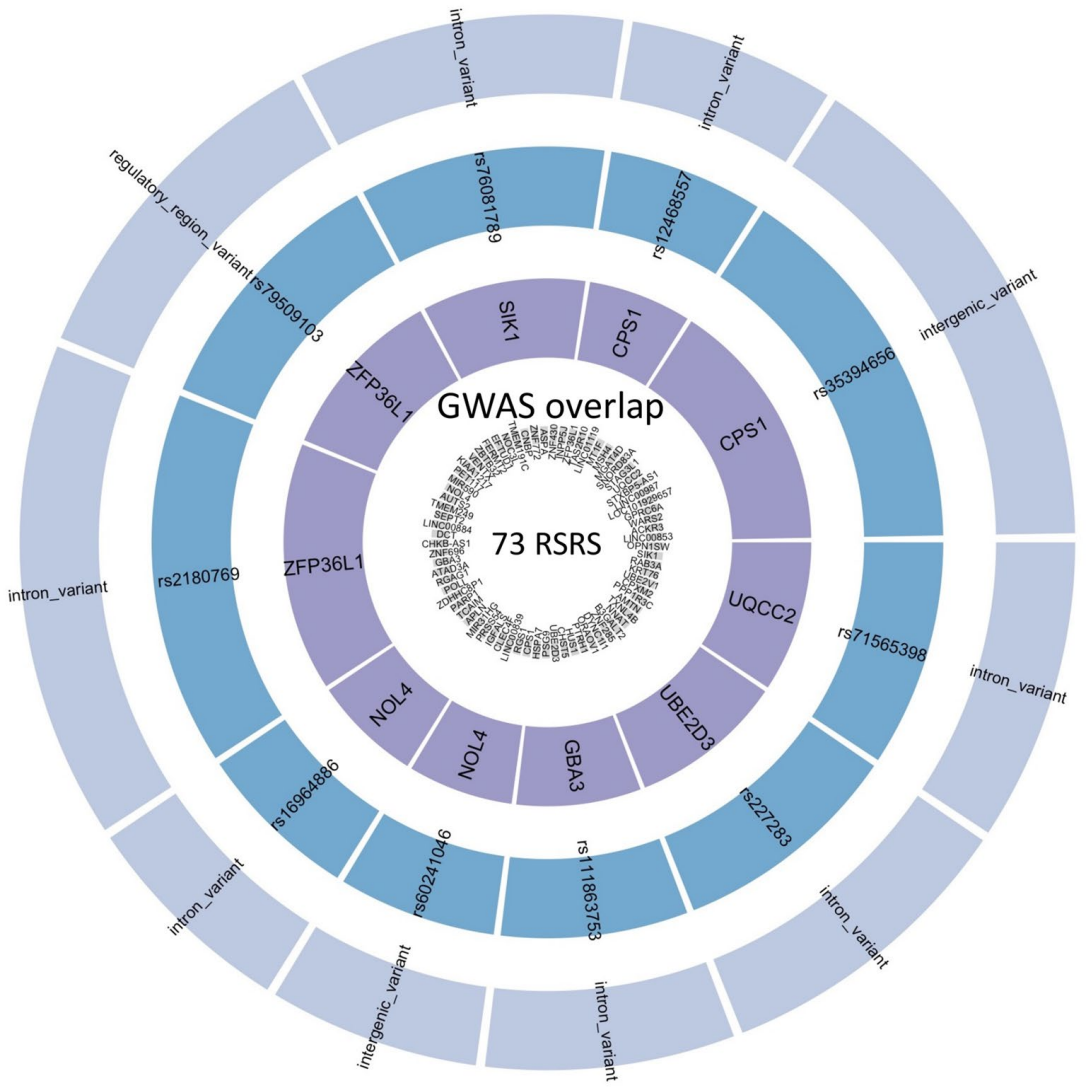
The overlap between the 73 RSRS genes and asthma genome wide association study catalog. The sector width for the SNP is proportional to the − log10 (adjusted p value) corresponding to each SNP.

Overlap between the 73 RSRS genes and asthma GWAS catalog is given in Fig. 6. There were seven genes identified by both RSRS and asthma GWAS catalog. Among these, each of gene CPS1 and gene NOL4 was mapped to 2 SNPs that belong to functional classes including the intronic variant and intergenic variant. Gene ZFP36L1 was mapped to 2 SNPs belonging to functional classes including the intronic variant and regulatory region variant, while each of SIK1, UQCC2, GBA3 and UBE2D3 was involved with one intronic SNP. Detailed information including p values are provided in Table S3.

### Connectivity map identifies potential asthma target signature

Using publicly available perturbagens, we identified drug targets related RSRS. The identified perturbagens (genetic or chemical) were primarily associated with immune function, cellular transport, regulation of transcription and inflammation. The perturbagens associated with asthma are shown in Table S4.

## Discussion

To the best of our knowledge, current risk prediction methods that are implemented using the combination of variants into polygenetic risk scores (PRS) did not take the opportunity to incorporate RNA-seq and demographic information. The current work filled this gap by constructing the RAN-seq analog of PRS, RNA-seq-based transcriptomic risk score (RSRS), based differential gene expression risk scores with adjustment for age and gender. In particular, we developed a novel 73-gene RNA-seq based score for predicting asthma risk by determining RSRS in a sample size of 695. We further validated the risk score in an independent validation set and obtained high predictive accuracy based on the AUC value. These findings demonstrate the RNA-seq based gene profiling score has the potential to support clinical diagnosis and to achieve satisfactory prediction accuracy for asthma.

Compared to PRSs, RSRSs have many advantages. Transcriptomics bridges the gap between genotype and phenotype, is physiologically closer to phenotype, and links genetic associations to biological mechanism. Therefore, RSRS should provide biologically tractable prediction that could potentially outperforms PRSs. Marigorta et al. showed that transcriptomic risk scores (TRSs) outperform PRSs in distinguishing Crohn’s disease from healthy samples^55^. Using data on 17 quantitative traits in UK Biobank, Liang et al. found that prediction accuracy of TRS was significantly higher than PRS in the African samples relative to the European reference set^56^. Other advantages of RSRS as a gene-level risk score include more biologically interpretable than SNP-level PRS, smaller and more manageable number of features, which in turn requires smaller samples to train prediction models^56^.

Several of the 73 RSRS genes were already linked with asthma. Leukocyte transcriptomes analysis from preschool children with acute wheeze identified genes including UBE2D3 involved that were significantly enriched in the innate immune responses^57^. Yucesoy et al.^58^ identified a novel Locus (18q12.1, NOL4) linked with diisocyanate-Induced occupational asthma via a genome-wide association study. Dysregulation of ZFP36L1/L2 in severe asthma epithelium was found to contribute to glucocorticoid non-responsiveness as well as epithelial barrier disruption^59^. The salt-inducible kinases (SIKs) are required for producing cytokines that regulate airway hyper-responsiveness, immune cell infiltration and inflammation^60^. Genome-wide significant loci (6p21.31, UQCC2) was identified by cross-trait meta-analysis associated with asthma and ADHD. Several top-ranking genetic perturbagens including PAK1, GSR, RBM15 and TNFRSF12A have been indicated by previous studies to be involved in allergy/asthma^61–63^. In particular, TNFRSF12A belongs to the family of Tumour necrosis factor (TNF) receptor. Current studies suggest that tumor necrosis factor (TNF)-α found in asthmatic airways, may directly alter the contractile properties of the airway smooth muscle and lead to the development of bronchial hyper-responsiveness^64^. Preliminary studies have demonstrated when treated with anti-TNF-α therapy, asthma patients showed an improvement in lung function, and airway hyperresponsiveness and a decrease in exacerbation frequency^65^. Our results also suggest several chemical perturbagens including mepacrine (cytokine production inhibitor) and dactolisib, WYE-125132, AZD-8055 (MTOR inhibitor). Cytokines are responsible for initiating the early stages of asthma and play a critical role in the persistence of the chronic inflammatory process in asthma because of many pro-inflammatory effect characteristics produced by cytokines^66,67^. Steroid-dependent asthma patients treated with Th2 cytokine inhibitor showed improvement in their pulmonary function and symptom control, and became less dependent on the inhaled corticosteroid^68^. Studies have also shown that patients experiencing an asthma attack showed significantly elevated serum MTOR pathway activation compared with patients in asthma remission, which suggests potential targets of MTOR inhibitor for treating asthma^69^. The results clearly demonstrated that the connectivity scores of perturbagens have role in RSRS. Our result also suggested the importance of age in predicting childhood asthma risk. Multiple studies have shown gender heterogeneity in the prevalence of asthma^70,71^. As children, boys have been consistently reported to have more asthma incidence than girls^70^, which was also confirmed in our study. As adults, compared with men, women exhibit an increased prevalence and severity of asthma^70^. Important factors in the gender heterogeneity related to asthma onset and severity include sex hormones, social and environmental factors, and responses to asthma therapeutics^70,72^. To examine the effect of gender-specific differences in changes of asthma prevalence, larger sample size and multi-omics should be investigated^70,71^.

Our study has some limitations. First, when the model includes several highly correlated variables, Lasso tends to pick only one or a few of them and shrinks the effect of the rest to 0^73^. For future studies, the elastic-net^74^ or adaptive Lasso^75^ may be adopted to alleviate the possible limitation of Lasso. Second, information provided in the public-domain gene expression data are limited and we only have access to the demographic information (age and gender) but no clinical data. However, even without clinical data, we were able to develop an RSRS with significant prediction accuracy for RNA-seq based data. One could anticipate when more demographic information, clinical parameters and symptoms (such as race, wheezing and polysensitization^22^) become available, the prediction power will be further improved. Third, the current datasets and sample sizes are somewhat small. Recently, efforts are made to generate big multi-omics data which could be incorporated in risk prediction. Further analysis of RNA-seq data with a large sample size taking social and environmental exposures into account will provide the opportunity to improve the accuracy of RSRS in predicting asthma risk.

In summary, using RNA-seq data, Logistic Lasso regression identified 73 gene-based RSRS that is predictive of asthma risk with AUC of 0.80. Our findings reveal the potential of RSRS for asthma risk and generated a new set of pathways and networks that may assist in defining genetic signatures linked with asthma. The pathways affected in our data can provide deeper insight in diseases mechanisms and to identify the most critical genes and drug or chemical that can be used to perturb this mechanism. Our RSRS method offers new statistical methodology to develop risk scores based on transcriptomic data in complex diseases.

## Supplementary Information

Supplementary Information.

## Data Availability

The data supporting this work is publicly available from NCBI GEO (Gene Expression Omnibus): https://www.ncbi.nlm.nih.gov/gds/?term=asthma.
