# Supplementary material for: Development and validation of an RNA-seq-based transcriptomic risk score for asthma
Source: Sci Rep. 2022 May 23;12:8643. doi: 10.1038/s41598-022-12199-0 (PMC9126925; doi:10.1038/s41598-022-12199-0)
Supplement: Supplementary file 1 — Supplementary Information. [file 41598_2022_12199_MOESM1_ESM.pdf]

Supplementary Information for
"**Development and Validation of an RNA-Seq-Based Transcriptomic Risk Score**"

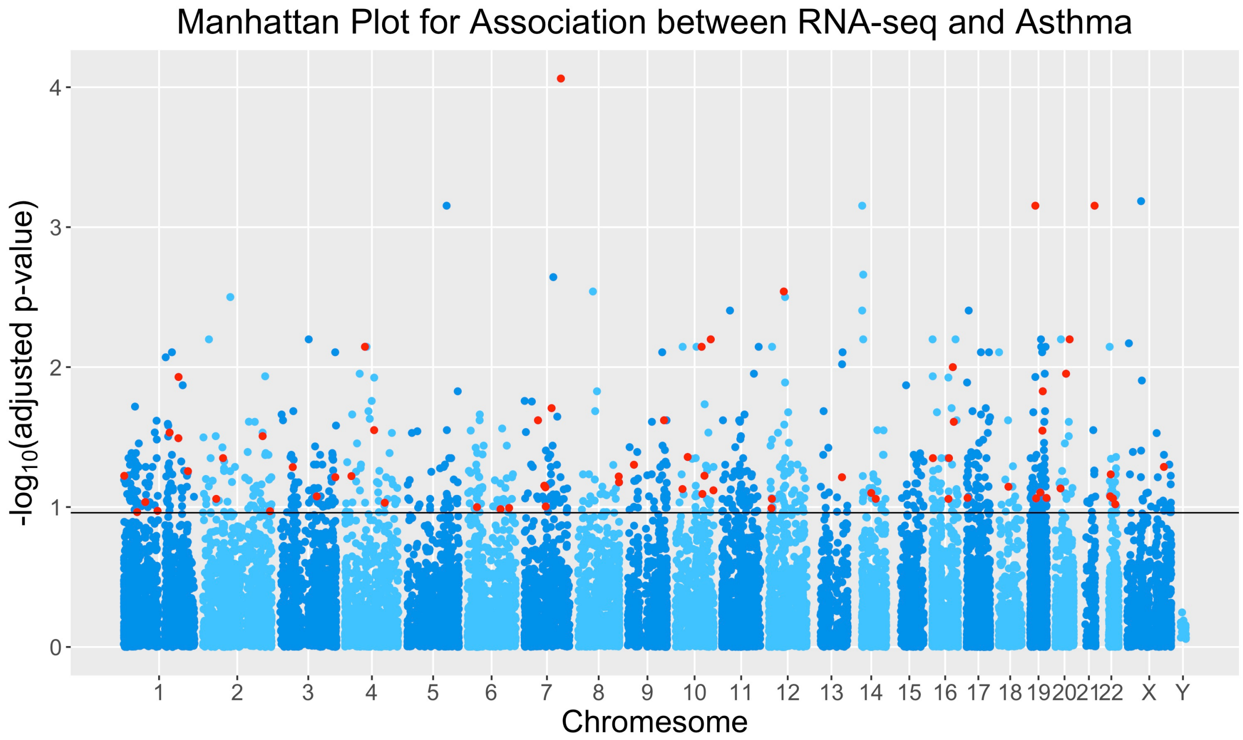

Supplementary Figure 1 (Figure S1): Manhattan plot for association between RNA-seq and asthma. The points beyond the black line represents the 1000 selected genes with the smallest adjusted p-values based on the DEG analysis. The red points correspond to the 73 RSRS genes.

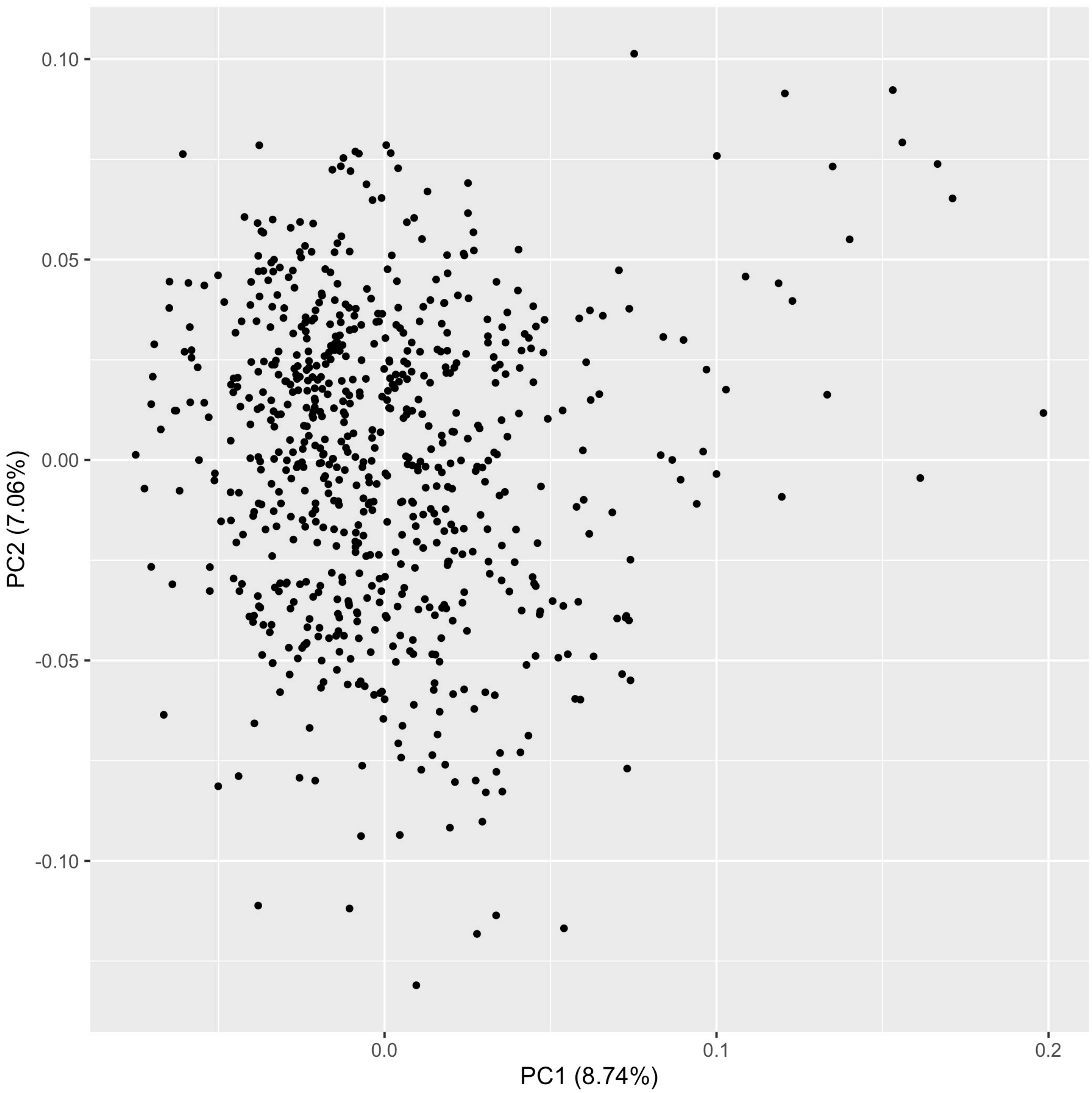

Supplementary Figure 2 (Figure S2): The visualization of Principal Component Analysis (PCA) among the normalized and log-transformed gene expression values of 73 genes.

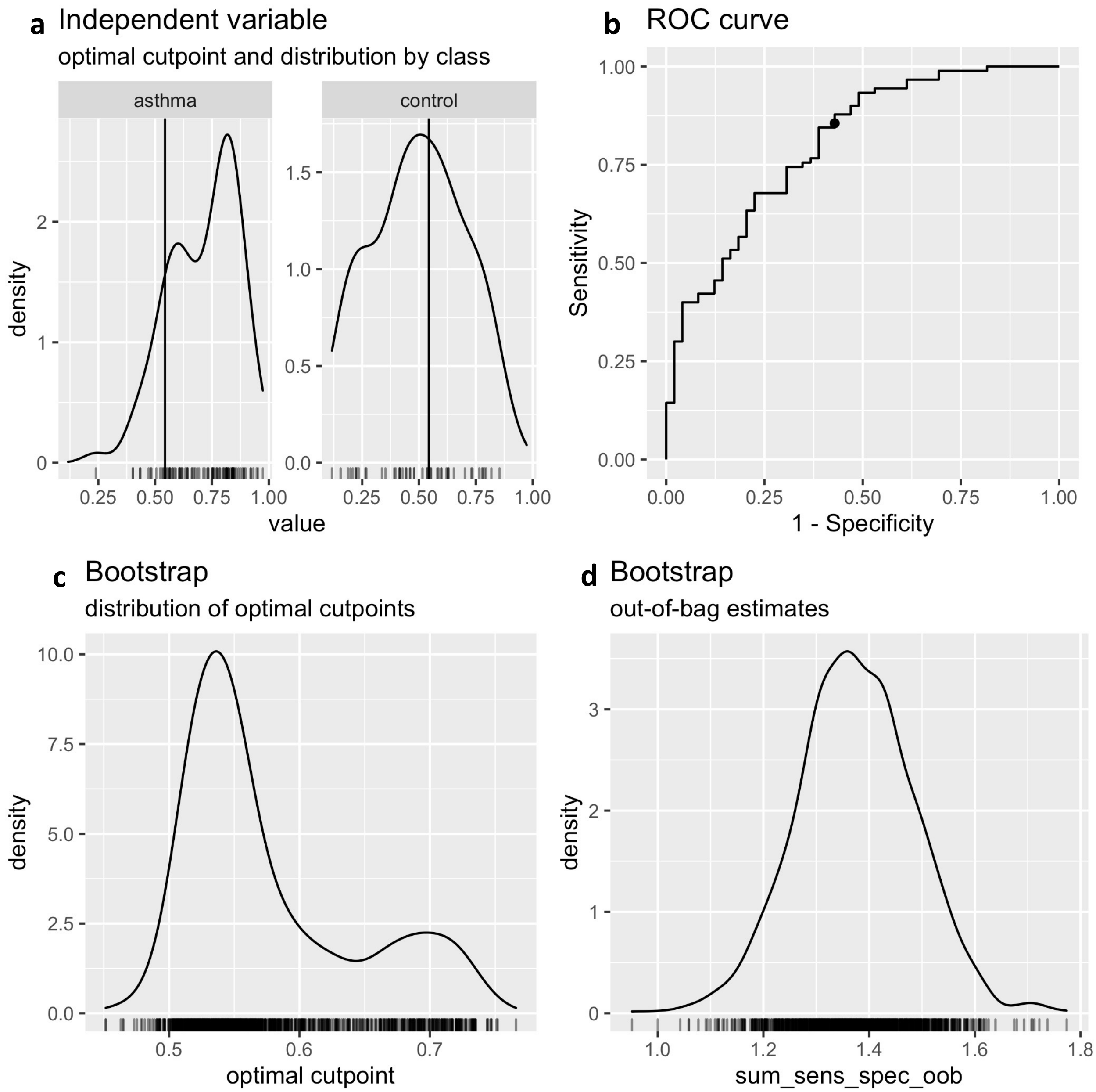

Supplementary Figure 3 (Figure S3): The distribution plots for the optimal cut point for the testing set of GSE152004. (**a**) Distribution plots of the response variables and the respective cutpoints for asthma and control groups; (**b**) the ROC curve with the optimal cutpoint displayed as a dot on the plot; (**c**) the bootstrapped distribution plot of optimal cutpoints; (**d**) the bootstrapped distribution plot of the out-of-bag metric values (sum of sensitivity and specificity).

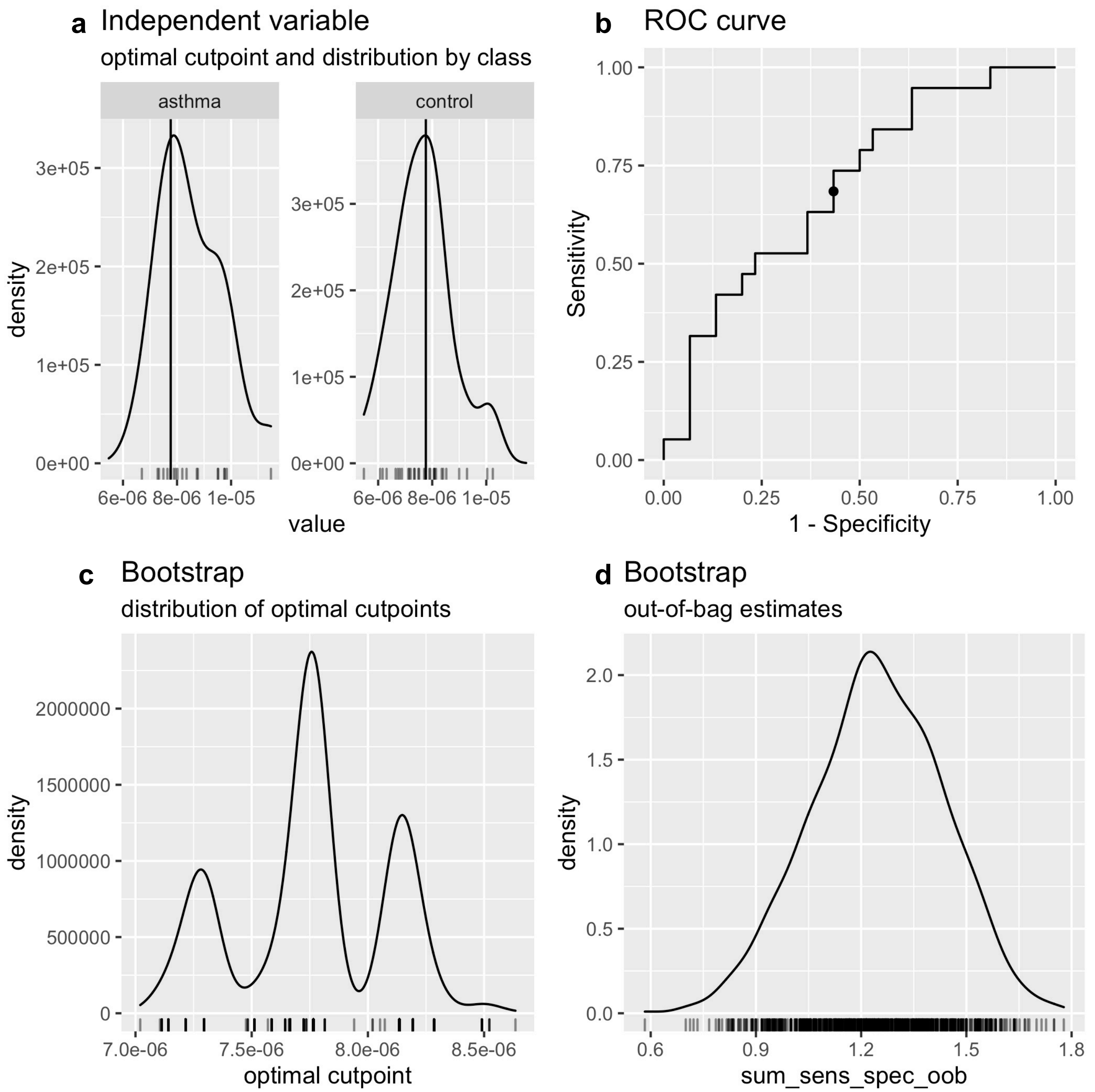

Supplementary Figure 4 (Figure S4): The distribution plots for the optimal cut point for the independent cohort GSE118761. (**a**) Distribution plots of the response variables and the respective cutpoints for asthma and control groups; (**b**) the ROC curve with the optimal cutpoint displayed as a dot on the plot; (**c**) the bootstrapped distribution plot of optimal cutpoints; (**d**) the bootstrapped distribution plot of the out-of-bag metric values (sum of sensitivity and specificity).

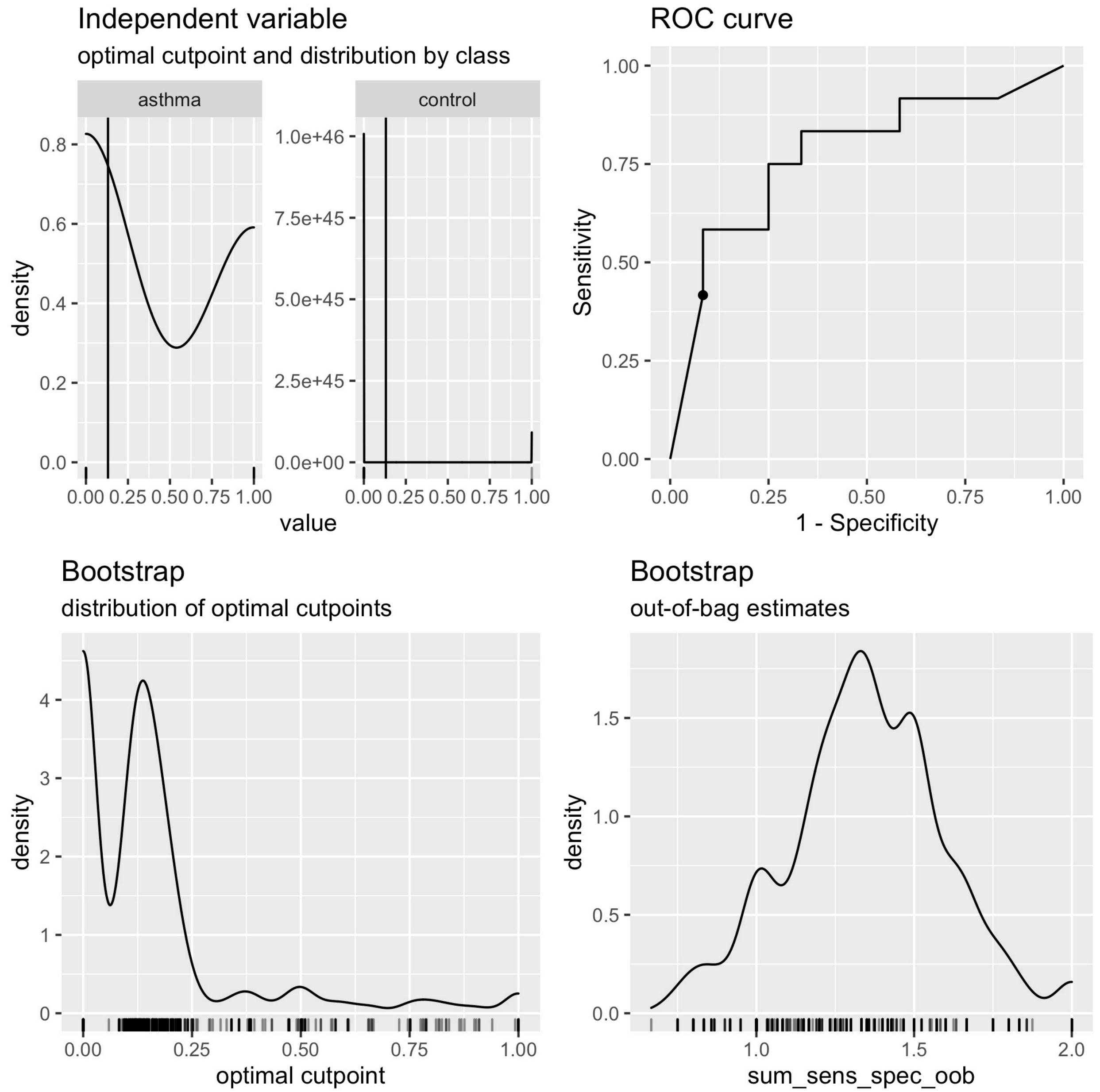

Supplementary Figure 5 (Figure S5): The distribution plots for the optimal cut point for the independent cohort GSE38003. (**a**) Distribution plots of the response variables and the respective cutpoints for asthma and control groups; (**b**) the ROC curve with the optimal cutpoint displayed as a dot on the plot; (**c**) the bootstrapped distribution plot of optimal cutpoints; (**d**) the bootstrapped distribution plot of the out-of-bag metric values (sum of sensitivity and specificity).

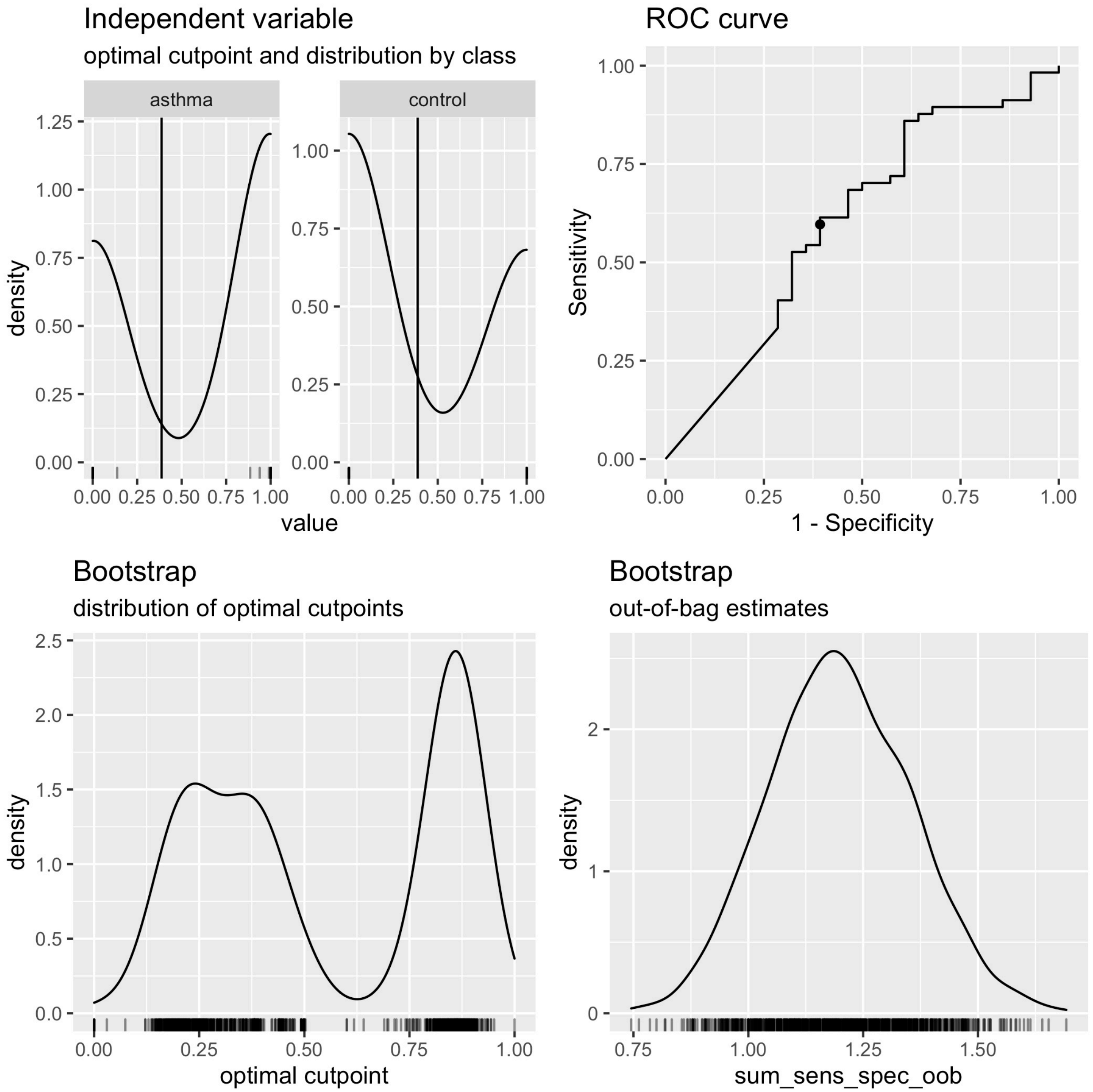

Supplementary Figure 6 (Figure S6): The distribution plots for the optimal cut point for the independent cohort GSE85567. (**a**) Distribution plots of the response variables and the respective cutpoints for asthma and control groups; (**b**) the ROC curve with the optimal cutpoint displayed as a dot on the plot; (**c**) the bootstrapped distribution plot of optimal cutpoints; (**d**) the bootstrapped distribution plot of the out-of-bag metric values (sum of sensitivity and specificity).

Network 1 : Xuan73GenesFinal - 2022-01-20 10:46 AM : Xuan73GenesFinal : Xuan73GenesFinal - 2022-01-20 10:46 AM

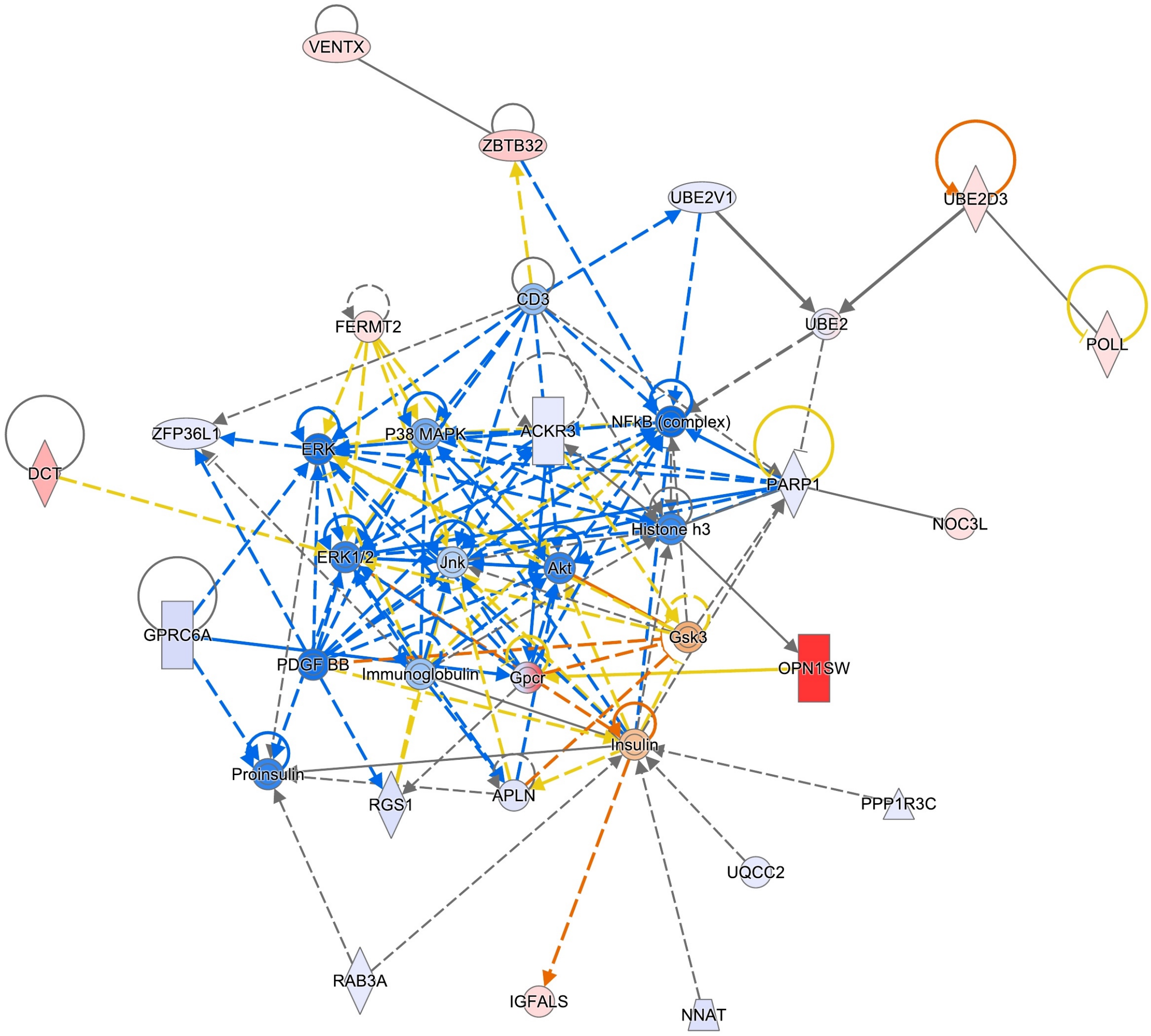

© 2000-2022 QIAGEN. All rights reserved.

Supplementary Figure 7 (Figure S7): RSRS genes pathway/network analyses. The Ingenuity Pathway Analyses methodology compares proportional representation of genes from a defined test set in a canonical pathway (a known, well-characterized pathway), compared to the proportional representation of the pathway genes in the entire set of known genes. The p-value is calculated using a right-tailed Fisher Exact test and indicates the likelihood of the pathway association under the random model.

Gene symbol	Gene name	log2 FC	padj	Weight	Odds ratio
OPN1SW	Opsin 1, Short Wave Sensitive	1.4549	1.00E-04	-0.0925	0.9116
SIK1	Salt Inducible Kinase 1	-0.337	7.00E-04	0.4641	1.5906
RAB3A	RAS Oncogene	-0.1843	7.00E-04	0.6721	1.9584
KRT76	Keratin 76	-1.7886	2.90E-03	0.0312	1.0317
UBE2V1	Ubiquitin Conjugating Enzyme E2 V1	-0.0701	6.30E-03	0.2621	1.2997
CPXM2	Carboxypeptidase X	-0.5161	6.30E-03	0.0986	1.1036
PPP1R3C	Protein Phosphatase 1 Regulatory Subunit 3C	-0.251	7.20E-03	0.0616	1.0636
AMTN	Amelotin	-0.5196	7.20E-03	0.0312	1.0317
TXNL4B	Thioredoxin Like 4B	0.0873	1.00E-02	-0.0693	0.9331
NNAT	Neuronatin	-0.3768	1.11E-02	0.0184	1.0185
B3GALT2	Beta-1,3-Galactosyltransferase 2	0.332	1.18E-02	-0.1126	0.8935
ZNF285	Zinc Finger Protein 285	0.387	1.49E-02	-0.036	0.9646
DYNC1I1	Dynein Cytoplasmic 1 Intermediate Chain 1	0.2338	1.96E-02	-0.2066	0.8134
ORAOV1	Oral cancer-overexpressed protein 1	0.0707	2.40E-02	-0.0254	0.9749
PTRH1	Peptidyl-TRNA Hydrolase 1 Homolog	-0.0786	2.40E-02	0.2897	1.336
HUS1	HUS1 Checkpoint Clamp Component	0.0526	2.40E-02	-0.4713	0.6242
CHST5	Carbohydrate Sulfotransferase 5	-0.2802	2.46E-02	0.0659	1.0681
UBE2D3	Ubiquitin Conjugating Enzyme E2 D3	0.0675	2.82E-02	-0.2919	0.7469
PSG9	Pregnancy Specific Beta-1-Glycoprotein 9	-0.5436	2.84E-02	0.2512	1.2855
HSPA7	Heat Shock Protein Family A (Hsp70) Member 7	0.3361	2.94E-02	-0.0359	0.9647
CPS1	Carbamoyl-Phosphate Synthase 1	0.31	3.11E-02	-0.2311	0.7937
RGS1	Regulator Of G Protein Signaling 1	-0.3817	3.22E-02	0.1439	1.1548
LINC00839	Long Intergenic Non-Protein Coding RNA 839	-0.5083	4.39E-02	0.1932	1.2131
CLEC4F	C-Type Lectin Domain Family 4	0.3551	4.47E-02	-0.0405	0.9603
IGFALS	Insulin Like Growth Factor Binding Protein Acid Labile Subunit)	0.251	4.47E-02	-0.0745	0.9282
PRSS54	Serine Protease 54	-0.2665	4.47E-02	0.1056	1.1114
MIR31HG	MIR31 Host Gene	0.143	4.99E-02	-0.0018	0.9982
APLN	Apelin	-0.3355	5.17E-02	0.1352	1.1447
TCAIM	T Cell Activation Inhibitor	0.0524	5.18E-02	-0.0206	0.9796
PARP1	Poly(ADP-Ribose) Polymerase 1	-0.0421	5.54E-02	0.0643	1.0664
ZDHHC8P1	Zinc Finger DHHC-Type Containing 8 Pseudogene 1	0.2271	5.84E-02	-0.0404	0.9604
POLL	DNA Polymerase Lambda	0.0443	5.97E-02	-0.7202	0.4867
RGAG1	Retrotransposon Gag Like 9	-0.1971	5.97E-02	0.1281	1.1366
ATAD3A	ATPase Family AAA Domain Containing 3A	-0.0619	5.99E-02	0.3976	1.4882
GBA3	Glucosylceramidase Beta 3	0.4443	6.01E-02	-0.0025	0.9975

ZNF696	Zinc Finger Protein 696	0.0708	6.05E-02	-0.5796	0.5601
CHKB-AS1	CHKB Divergent Transcript	0.1242	6.11E-02	-0.1857	0.8305
DCT	Dopachrome Tautomerase	0.561	6.12E-02	-0.0283	0.9721
LINC00884	long intergenic non-protein coding RNA 884	-0.1264	6.12E-02	0.2673	1.3064
2-Sep	Septin 2	0.0408	6.44E-02	-0.2553	0.7747
TMEM249	Transmembrane Protein 249	0.2923	6.66E-02	-0.0238	0.9764
AUTS2	Autism Susceptibility Gene 2 Protein	-0.0635	7.04E-02	1.7257	5.6167
NOL4	nucleolar protein 4	0.8959	7.16E-02	-0.1208	0.8862
MIR590	MicroRNA 590	0.2539	7.22E-02	-0.003	0.997
PET117	PET117 Cytochrome C Oxidase Chaperone	-0.0596	7.36E-02	1.8114	6.1189
KIAA1217	KIAA1217	-0.0577	7.44E-02	0.1184	1.1257
VENTX	VENT Homeobox	0.2444	7.60E-02	-0.056	0.9455
ZBTB32	Zinc Finger And BTB Domain Containing 32	0.384	7.88E-02	-0.0211	0.9791
FERMT2	Fermitin family homolog 2	0.1387	7.93E-02	-0.3573	0.6996
EFTUD1	Elongation Factor Like GTPase 1	-0.0457	8.04E-02	1.139	3.1236
NOC3L	Nucleolar complex protein 3 homolog	0.0727	8.06E-02	0.1965	1.2171
TMEM191C	Transmembrane Protein 191C	-0.1639	8.38E-02	0.4606	1.585
CNBP	CCHC-Type Zinc Finger Nucleic Acid Binding Protein	0.0358	8.40E-02	-0.7418	0.4763
ZNF772	Zinc Finger Protein 772	0.0949	8.61E-02	-0.3121	0.7319
ASPA	Aspartoacylase	0.4227	8.61E-02	-0.1869	0.8296
ZNF430	Zinc Finger Protein 430	0.0614	8.67E-02	-0.2838	0.7529
INPP5J	Inositol Polyphosphate-5-Phosphatase J	0.1221	8.67E-02	-0.1478	0.8626
ZFP36L1	Zinc finger protein 36, C3H type-like 1	-0.0741	8.75E-02	0.0775	1.0805
TAS2R10	Taste 2 Receptor Member 10	0.5081	8.75E-02	-0.0348	0.9658
LINC01119	long intergenic non-protein coding RNA 1119	-0.2405	8.75E-02	0.1015	1.1068
MT1F	Metallothionein 1F	-0.1399	8.75E-02	0.0476	1.0488
MSH4	MutS Homolog 4	0.3791	9.21E-02	-0.1195	0.8874
MGAT4D	MGAT4 Family Member D	0.539	9.31E-02	-0.1797	0.8355
SNORD83A	Small Nucleolar RNA, C/D Box 83A	0.3374	9.56E-02	-0.0193	0.9809
STAG3L1	Stromal Antigen 3-Like 1	0.1651	9.91E-02	-0.1189	0.8879
UQCC2	Ubiquinol-Cytochrome C Reductase Complex Assembly Factor 2	-0.0676	1.00E-01	0.1733	1.1892
STXBP5-AS1	STXBP5 Antisense RNA 1	-0.2382	1.02E-01	0.1155	1.1225
LINC00987	Long Intergenic Non-Protein Coding RNA 987	0.1704	1.02E-01	-0.1094	0.8964
LOC101929657	Uncharacterized LOC101929657	-0.1837	1.02E-01	0.1434	1.1542
GPRC6A	G Protein-Coupled Receptor Class C Group 6 Member A	-0.4579	1.04E-01	0.0327	1.0333

WARS2	Tryptophanyl TRNA Synthetase 2	0.0629	1.07E-01	-0.44	0.644
ACKR3	Atypical chemokine receptor 3	-0.1574	1.07E-01	0.3931	1.4815
LINC00853	long intergenic non-protein coding RNA 853	0.1355	1.09E-01	-0.0945	0.9098

Supplementary Table 1 (Table S1): The 73 genes identified by Lasso together with gene names, log2 fold changes (Log2 FC), adjusted p values (p_{adj}), weights (regression coefficients) and odds ratios with respect to one unit change in the normalized and log-transformed gene expression level.

IPA categories		
Top Canonical Pathways	[a]p-value	[b]Overlap
Eumelanin Biosynthesis	1.02E-02	25.0%
Lactose Degradation III	1.28E-02	20.0%
Urea Cycle	1.53E-02	16.7%
Hypoxia Signaling in the Cardiovascular System	1.63E-02	2.6%
Apelin Adipocyte Signaling Pathway	2.29E-02	2.2%
Top Associated Network Functions	[c]Score	
Carbohydrate Metabolism, Cell-To-Cell Signaling and Interaction, Cellular Compromise	47	
Cell Cycle, DNA Replication, Recombination, and Repair, Reproductive System Development and Function	33	
Connective Tissue Development and Function, Nervous System Development and Function, Skeletal and Muscular System Development and Function	27	
Cellular Development, Cellular Growth and Proliferation, Cellular Movement	17	
Developmental Disorder, Neurological Disease, Organismal Injury and Abnormalities	2	
Cell-To-Cell Signaling and Interaction, Cellular Assembly and Organization, Cellular Response to Therapeutics	2	

Supplementary Table 2 (Table S2): Top pathways and network functions identified for 73 RSRS genes. a: The p-value indicates the probability of association of molecules from our 73 RSRS genes with the canonical pathway by random chance alone using Fisher's exact test. b: In a given pathway, the overlap ratio is calculated as number of genes in our dataset that meet the cutoff criteria, divided by the total number of genes involved in that pathway. c: Networks with scores ≥2 have a 99.9% confidence of not being generated by random chance.

PUBMEDID	MAPPED_TRAIT	CHR_ID	CHR_POS	MAPPED_GENE	SNPS	CONTEXT	P-VALUE
31619474	attention deficit hyperactivity disorder, asthma	6	33633953	UQCC2	rs71565398	intron_variant	4.00E-08
31619474	attention deficit hyperactivity disorder, asthma	4	102686096	UBE2D3	rs227283	intron_variant	3.00E-08
29551627	cortisol measurement, response to corticosteroid, response to synacthen	4	22794376	GBA3	rs111863753	intron_variant	8.00E-07
25918132	response to diisocyanate, asthma	18	33810904	NOL4	rs60241046	intergenic_variant	7.00E-06
25918132	response to diisocyanate, asthma	18	33742030	NOL4	rs16964886	intron_variant	5.00E-06
31959851	asthma	14	68332385	ZFP36L1	rs2180769	intron_variant	7.00E-13
34594039	asthma	14	68844624	ZFP36L1	rs79509103	regulatory_region_variant	3.00E-09
29083406	allergy	21	43426546	SIK1	rs76081789	intron_variant	1.00E-08
23517042	asthmatic body mass index	2	210630863	CPS1	rs12468557	intron_variant	9.00E-06
31669095	asthmatic body mass index	2	210759245	CPS1	rs35394656	intergenic_variant	4.00E-13

Supplementary Table 3 (Table S3): The overlap between 73 RSRS genes and asthma GWAS catalog.

Rank	Score	Type	ID	Name	Description
1	99.14	kd	CGS001-1504	CTRB1	-
2	98.61	oe	ccsbBroad304_01144	PAK1	PAKA subfamily
3	98.37	kd	CGS001-3421	IDH3G	-
4	98.32	kd	CGS001-7508	XPC	-
5	98.28	kd	CGS001-2936	GSR	Oxidoreductases
6	98.05	kd	CGS001-64783	RBM15	RNA binding motif (RRM) containing
7	98.04	kd	CGS001-23659	PLA2G15	-
8	97.41	cp	BRD-K86003836	flubendazole	Tubulin inhibitor
9	97.02	kd	CGS001-9183	ZW10	-
10	96.96	kd	CGS001-80306	MED28	-
11	96.95	kd	CGS001-23038	WDTC1	DDB1 and CUL4 associated factors
12	96.88	oe	ccsbBroad304_07616	ZNF238	-
13	96.08	kd	CGS001-5829	PXN	-
14	95.95	kd	CGS001-5184	PEPD	Methionyl aminopeptidase
15	95.7	cp	BRD-K76674262	homoharringtonine	Protein synthesis inhibitor
16	95.59	kd	CGS001-55604	LRRC16A	-
17	95.56	kd	CGS001-7126	TNFAIP1	BTB/POZ domain containing
18	95.46	kd	CGS001-3094	HINT1	-
19	95.31	cp	BRD-A45889380	mepacrine	Cytokine production inhibitor
20	94.95	kd	CGS001-7533	YWHAH	-
21	94.76	kd	CGS001-51330	TNFRSF12A	Tumour necrosis factor (TNF) receptor family
22	94.68	kd	CGS001-5327	PLAT	Chymotrypsin
23	94.52	kd	CGS001-10250	SRRM1	-
24	94.27	kd	CGS001-5328	PLAU	Chymotrypsin
25	93.78	kd	CGS001-26353	HSPB8	Heat shock proteins / HSPB
26	93.76	cp	BRD-A25687296	emetine	Protein synthesis inhibitor
27	93.61	cp	BRD-K85985071	ellipticine	Topoisomerase inhibitor
28	93.51	kd	CGS001-10733	PLK4	Polo-like kinase (PLK) family
29	93.48	cp	BRD-K12184916	dactolisib	MTOR inhibitor
30	93.43	cp	BRD-A45498368	WYE-125132	MTOR inhibitor
31	93.29	oe	ccsbBroad304_00937	LGALS2	Lectins, galactoside-binding
32	92.9	kd	CGS001-8848	TSC22D1	-
33	92.59	cp	BRD-K69932463	AZD-8055	MTOR inhibitor
34	92.5	kd	CGS001-5781	PTPN11	Protein tyrosine phosphatases
35	92.31	cp	BRD-K80348542	cephaeline	Protein synthesis inhibitor
36	91.92	kd	CGS001-8678	BECN1	-
37	91.71	kd	CGS001-375346	TMEM110	-
38	91.71	kd	CGS001-5525	PPP2R5A	Serine/threonine phosphatases / Protein phosphatase 2, regulatory subunits
39	91.04	cp	BRD-K94294671	OSI-027	MTOR inhibitor
40	91.01	cp	BRD-A02481876	importazole	Importin-beta transport receptor inhibitor
41	90.92	kd	CGS001-2853	GPR31	GPCR / Class A : Orphans

42	90.92	kd	CGS001-831	CAST	-
43	90.9	kd	CGS001-54566	EPB41L4B	-
44	90.8	kd	CGS001-2677	GGCX	Carboxylases
45	90.74	kd	CGS001-9159	PCSK7	Subtilisin
46	90.59	kd	CGS001-9016	SLC25A14	Mitochondrial uncoupling proteins
47	90.52	kd	CGS001-5478	PPIA	Cis-trans-isomerases
48	90.47	cp	BRD-K76064317	tyrphostin-AG-1296	FLT3 inhibitor
49	90.34	kd	CGS001-51015	ISOC1	-
50	90.26	kd	CGS001-2335	FN1	Endogenous ligands
51	90.06	cp	BRD-U33728988	QL-X-138	MTOR inhibitor
52	90.03	oe	ccsbBroad304_00832	IFNB1	Interferons
53	89.93	kd	CGS001-54499	TMCO1	-
54	89.6	kd	CGS001-8022	LHX3	Homeoboxes / LIM class
55	89.46	kd	CGS001-6428	SRSF3	RNA binding motif (RRM) containing
56	89.39	kd	CGS001-5887	RAD23B	-
57	89.27	kd	CGS001-255738	PCSK9	Subtilisin
58	89.22	kd	CGS001-4143	MAT1A	-
59	89.16	kd	CGS001-6129	RPL7	L ribosomal proteins
60	89.15	kd	CGS001-50865	HEBP1	Endogenous ligands
61	89.11	kd	CGS001-6927	HNF1A	Homeoboxes / HNF class
62	88.9	kd	CGS001-598	BCL2L1	Serine/threonine phosphatases / Protein phosphatase 1, regulatory subunits
63	88.72	kd	CGS001-5021	OXTR	GPCR / Class A : Vasopressin and oxytocin receptors
64	88.58	kd	CGS001-134187	POU5F2	Homeoboxes / POU class
65	88.36	kd	CGS001-4869	NPM1	-
66	88.31	cp	BRD-K44497846	enalapril	ACE inhibitor
67	88.27	cp	BRD-K94325918	kinetin-riboside	Apoptosis stimulant
68	88.08	kd	CGS001-124274	GPR139	GPCR / Class A : Orphans
69	88.06	oe	ccsbBroad304_00763	HNF4A	Hepatocyte nuclear factor-4 receptors
70	88.05	cp	BRD-A26002865	verrucarin-a	Protein synthesis inhibitor
71	87.69	cp	BRD-K32828673	chelidonine	Tubulin inhibitor
72	87.62	oe	ccsbBroad304_00034	AES	-
73	87.19	kd	CGS001-64343	AZI2	-
74	87.17	kd	CGS001-89884	LHX4	Homeoboxes / LIM class
75	87.06	kd	CGS001-6309	SC5DL	-
76	86.48	cp	BRD-K37194137	III606050	Cytochrome P450 inhibitor
77	86.44	cp	BRD-K91370081	anisomycin	DNA synthesis inhibitor
78	86.41	kd	CGS001-10652	YKT6	-
79	86.14	kd	CGS001-1212	CLTB	-
80	86.09	kd	CGS001-1969	EPHA2	Type XIII RTKs: Ephrin receptor family
81	86	kd	CGS001-4771	NF2	A-kinase anchor proteins
82	85.76	cp	BRD-A92537424	danazol	Estrogen receptor antagonist
83	85.49	kd	CGS001-435	ASL	-

8535	-85.03	kd	CGS001-9052	GPRC5A	GPCR / Class C : Orphans
8536	-85.26	kd	CGS001-55143	CDCA8	-
8537	-88.67	cp	BRD-K31987754	oleylethanolamide	Cannabinoid receptor agonist
8538	-89.78	kd	CGS001-6713	SQLE	Lanosterol biosynthesis pathway
8539	-89.81	kd	CGS001-23523	CABIN1	-
8540	-90.53	kd	CGS001-6046	BRD2	Bromodomain kinase (BRDK) family
8541	-90.82	kd	CGS001-7158	TP53BP1	-
8542	-91.28	kd	CGS001-55872	PBK	TOPK family
8543	-91.33	kd	CGS001-9989	PPP4R1	Serine/threonine phosphatases / Protein phosphatase 4, regulatory subunits
8544	-91.38	kd	CGS001-224	ALDH3A2	Aldehyde dehydrogenases
8545	-91.77	oe	ccsbBroad304_07078	TSPAN6	Tetraspanins
8546	-92.06	kd	CGS001-55012	PPP2R3C	Serine/threonine phosphatases / Protein phosphatase 2, regulatory subunits
8547	-93.89	kd	CGS001-51311	TLR8	Toll-like receptor family
8548	-93.93	kd	CGS001-83873	GPR61	GPCR / Class A : Orphans
8549	-93.99	kd	CGS001-81550	TDRD3	Tudor domain containing
8550	-94.59	kd	CGS001-23585	TMEM50A	-
8551	-94.65	kd	CGS001-2629	GBA	-
8552	-97.23	kd	CGS001-10950	BTG3	-
8553	-97.32	kd	CGS001-10196	PRMT3	Protein arginine N-methyltransferases
8554	-98.08	kd	CGS001-6397	SEC14L1	-
8555	-98.35	cp	BRD-K39569857	avrainvillamide-analog-3	nucleophosmin inhibitor
8556	-98.51	kd	CGS001-10094	ARPC3	Actin related protein 2/3 complex subunits
8557	-98.64	kd	CGS001-6856	SYPL1	-
8558	-98.89	kd	CGS001-9352	TXNL1	-
8559	-99.89	kd	CGS001-1871	E2F3	-

Supplementary Table 4 (Table S4): Description of connected perturbagens relevant for 73 RSRS in asthma. Type (based on biological / chemical function drug), location and entrez names of genetic perturbagens have been shown. One hundred and eight perturbagens associated with 73 RSRS are listed here.
